# COMPASSION-BASED ENVIRONMENTAL VOLUNTEERISM AMONG OLDER ADULTS: A CASE STUDY OF TZU CHI RECYLING PROGRAM

**DOI:** 10.1093/geroni/igz038.2690

**Published:** 2019-11-08

**Authors:** Hsinyi Hsiao, Chiu-Tien Hsu, Lei Chen, Jinli Wu, Pao-Sheng Chang, Chin-Lon Lin, Miing-Nan Lin, Tin-Kwang Lin

**Affiliations:** 1 Tzu Chi University, Hualien, Taiwan, Taiwan; 2 National Chung Chen University, Chiayi, Chiayi, Taiwan (Republic of China); 3 University of California, Los Angeles, Los Angeles, California, United States; 4 University of Southern California, Los Angeles, California, United States; 5 Hualien Tzu Chi Hospital, Hualien 970, Hualien, Taiwan (Republic of China); 6 Dalin Tzu Chi Hospital, Dalin, Chiayi, Taiwan (Republic of China)

## Abstract

To promote environmental sustainability and mitigate climate change that causes numerous families to suffer from natural disasters, a group of older residents’ volunteer to recycle usable materials by setting up recycling stations in their communities and transform recycled bottles into eco-friendly blankets for disaster survivors globally. This study examined long-term effects of the peer-led Tzu Chi Recycling Program (TCRP) on older adults’ compassion, psychological and physiological well-being. Using a quasi-experimental design, 1-year longitudinal data were collected from older adults at recycling stations (intervention group n = 36) and community centers (control group n = 36) in rural areas in Southern Taiwan. Findings from two-way repeated analysis of variance show that TCRP significantly improved older adults’ self-compassion, compassion for others, depression, hostility, happiness, and hypertension. Older adults built resilience through environmental volunteering in the TCRP as an environmental sustainability model for health promotion and social good

